# Segmentation of Change in Surface Geometry Analysis for Cultural Heritage Applications

**DOI:** 10.3390/s21144899

**Published:** 2021-07-19

**Authors:** Sunita Saha, Jacek Martusewicz, Noëlle L. W. Streeton, Robert Sitnik

**Affiliations:** 1 Institute of Micromechanics and Photonics, Faculty of Mechatronics, Warsaw University of Technology, ul. Św. Andrzeja Boboli 8, 02-525 Warsaw, Poland; Sunita.saha@pw.edu.pl; 2 Faculty of Conservation and Restoration of Works of Art, Academy of Fine Arts in Warsaw, ul. Krakowskie Przedmieście 5, 00-068 Warszawa, Poland; jacek.martusewicz@asp.waw.pl; 3 Department of Archaeology, Conservation & History, University of Oslo, P.O. Box 1072 Blindern, 0316 Oslo, Norway; n.l.w.streeton@iakh.uio.no

**Keywords:** imaging, monitoring, 3D data, geometric change, restoration, segmentation, visualization

## Abstract

This work proposes a change-based segmentation method for applications to cultural heritage (CH) imaging to perform monitoring and assess changes at each surface point. It can be used as a support or component of the 3D sensors to analyze surface geometry changes. In this research, we proposed a new method to identify surface changes employing segmentation based on 3D geometrical data acquired at different time intervals. The geometrical comparison was performed by calculating point-to-point Euclidean distances for each pair of surface points between the target and source geometry models. Four other methods for local distance measurement were proposed and tested. In the segmentation method, we analyze the local histograms of the distances between the measuring points of the source and target models. Then the parameters of these histograms are determined, and predefined classes are assigned to target surface points. The proposed methodology was evaluated by considering two different case studies of restoration issues on CH surfaces and monitoring them over time. The results were presented with a colormap visualization for each category of the detected change in the analysis. The proposed segmentation method will help in the field of conservation and restoration for the documentation and quantification of geometrical surface change information. This analysis can help in decision-making for the assessment of damage and potential prevention of further damage, and the interpretation of measurement results.

## 1. Introduction

There are two main approaches to surface geometry segmentation: geometry (single geometry representation based) and change segmentation (two or more states represented by corresponding geometries). The single geometry segmentation implies the detection and grouping of similarly shaped objects’ parts, while change segmentation is the grouping of likewise change behavior parts of the data. The change analysis of data from surfaces on CH objects involves the comparison of two or more 3D geometries to assess the global statistical information [1,2]. This is essential to have a brief overview of the changes, which might be related to natural aging and deformation, as well as to surface alterations and the formation of crusts, purposeful destruction, or the addition/removal of material during restoration. This information can be assessed by simply comparing the two geometries and their different parameters. In Reference [3], the change detection was performed by using the global geometry comparison method of cloud to mesh (C2M), where they lost information while creating the mesh for the compared surface. Later a direct point-to-point geometry comparison was developed in Reference [4], which is a model-to-model cloud comparison (M3C2), and applied for change detection in References [1,5]. The method could identify the loss and deposit on the surface irrespective of noise and unsmooth region. These works were dedicated to CH change detection over time; however, they were conducted by using a physical marker for the reference. The use of a physical marker is prohibited for various CH objects. Moreover, the global geometry comparison alone is sensitive to the cross-time registration error of the two models. In our approach, we overcome the registration error based on the known unchanged part of the surface and use of physical marker for the change detection. However, it would be beneficial if the global change information could be identified and grouped based on the behavior of the change of local geometry of each point on the surface.

There are many approaches to segmentation and automation for one-phase geometry identification dedicated to CH [6,7,8], using several geometric rules. The scanning of real objects is affected by various problems that make it difficult to perform the segmentation without uncertainty. The common factors that produce ambiguity in the recognition process of classification are point location noise [9,10,11], mostly from the uneven part of the surface, the thermal noise of CCD/CMOS detectors, optical phenomena [12], and the coarse representation of continuous surfaces due to triangular approximations [13]. The most common approaches for the geometric segmentation process are edge-based (curvature), region-based (density, smoothness, similarity), model-based, and hybrid methods, where both edge-based and region-based segmentation are used [14]. The use of machine learning approaches makes the method more robust to the overall noise, as well as region noise and occlusions [7,10,11,15,16]. However, there is no required segmentation functionality defined in the application to CH change measurements. Change-based segmentation is an attempt to identify and classify the behavioral similarity of the change occurrence and assign it a linguistic attribute for a portion of the surface [17]. However, in the field of preservation and documentation of CH objects, information can be collected in various ways like 2D RGB, Multispectral Imaging, infrared, 3D information, Reflection Transformation Imaging or combining 3D with RGB or multispectral [18], according to the analysis needed and the selection of data accumulator, irrespective of the size of the data [5]. The change-based segmentation can be obtained from 3D scanning of an object’s surface and can be localized to detect and calculate surface geometry changes over the monitoring period [19]. To highlight gaps in knowledge, with specific reference to CH research, the analytical approaches of geometry segmentation are presented based on geometric analysis of an object’s surface in general 3D application fields. The study was performed on the previously developed one-phase geometry segmentation methods and tested to incorporate the same with our proposed segmentation dedicated to the change measurement in CH over time.

This paper proposes a novel change-based segmentation method of CH surfaces that could support 3D sensors with additional functionality. This method assesses changes between the target and source geometry models captured at different time intervals. It is based on analysis of the local distance’s histograms between the measuring points of the source and target models. Then the certain parameters of the histograms are determined, and predefined classes are assigned to target surface points. The overall data-processing chain is composed of four major processing stages, i.e., reconstruction of the 3D models, global and local geometry analysis, segmentation, and visualization. The analysis of the collected data and tracking down changes in CH objects over time (from different aspects, such as humidity, rainfall, snowfall, climate change, etc.) involves a set of problems for which the proposed method could provide detailed information on a 3D surface. In addition, it can help conservators and scientists in the documentation with the 3D digital representation involved in their work. The work is also focused on developing visualization of the results obtained on the surface to make it a more user-friendly representation of detected changes. The goal of the work is in line with the application of optical sensing techniques to CH, monitoring and grouping changes on the surface of CH objects before and after conservation.

## 2. Methodology

To further improve the digital documentation of change on CH objects, we propose a segmentation method to recognize the several different possible states of changes locally on the surface. The goal of this work is to develop an automated change-detection method to retrieve various behaviors of local geometry for each point change from the CH surface. To do this, we considered calculating the distances between each point from the target surface to the source surface. However, for that, accurate point-to-point mapping between the two stages of data is a crucial step to calculate the actual value of the change. In this work, a corresponding changed point search algorithm was developed in four stages, considering the direction of the normal vector of the target surface point and the displacement vector from the target to the source surface point. The Euclidian distance was calculated in four different ways, from each point on the target surface to the corresponding changed point on the source surface, to analyze its influence on the segmentation results. For each surface point, a local distance histogram was stored, and certain parameters were calculated. From the calculated parameters, the identification of each surface point was performed and stored in a separate category. Based on the expertise of the conservators, each detected category was inspected and named locally, based on the restoration/conservation and monitoring over time. In this work, we also considered developing the visualization of the results, i.e., a representation of colors for the types of change on the surface, to make it a more user-friendly representation of the detected changes, based on the histogram behavior. In this section, a detailed description of the four major phases of the entire work, as introduced above, is given.

### 2.1. Reconstruction

In this section, the 3D modeling of the object is composed of two phases, i.e., stitching of the filtered point clouds to reconstruct the 3D model and cross-time alignment of more than one model.

#### 2.1.1. Stitching of Point Clouds

**Figure 1:**
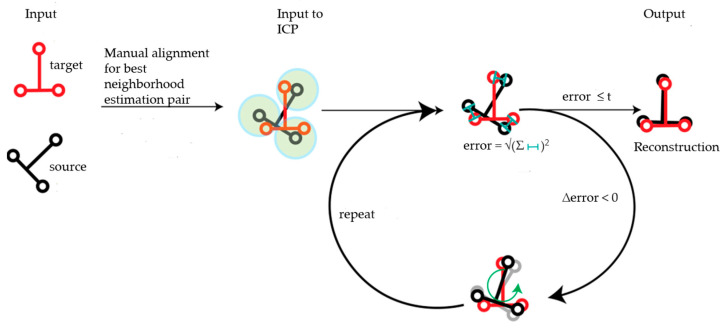
Sketch of the reconstruction of the 3D model.

To reconstruct the 3D model for the selected object, each of the filtered point clouds was stitched together in the real coordinate space concerning a target point cloud (*T*_0_). For the rest of the source scans (*T*_1*… n*_), a manual alignment close to the target point cloud was introduced for a rough estimation of the 3D model of the object from the user perspective view. Then stitching of the scans was performed by using the optimized iterative closest point iteration (ICP) algorithm [20] for each pair of point clouds. ICP was recursively performed as shown in Figure 1 for each pair of consecutive point clouds until the minimum error of the algorithm was reached. A condition was set for the number of iterations to the best fit: the calculated root mean square (RMS) of the ICP algorithm ≤Threshold value (*t*). The average point-to-point distance of target was considered as the threshold value for this analysis. The global transformation induced in the source point cloud was stored to get the final 3D model of the entire object’s surface. For the next run, *T*_1_ was considered the target point cloud, and the source scans are updated as *T*_2*… n*_.

#### 2.1.2. Cross-Time Alignment of the Models

**Figure 2:**
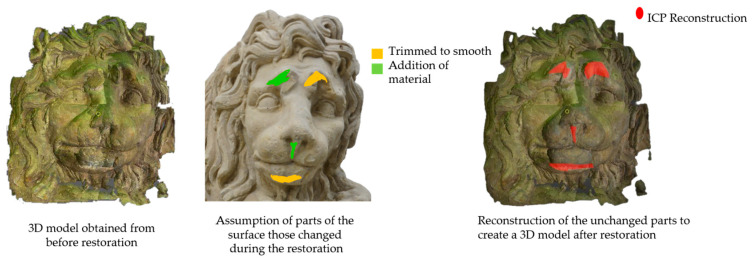
Three-dimensional model of the face of the ‘lying lion’; sandstone; 150 × 71 × 86 cm; owner: Radziwiłł Palace in Nieborów; possible solution to avoid alignment error for the face (~30 × 35 cm) in case of known reference of no change.

The cross-time alignment of the data from two different phases, i.e., before and after change, in terms of variation in size of the data is a global problem. However, to compare the geometry over time, the 3D models obtained from the two measurements of the same object must be aligned in the same 3D space. By following the same ICP algorithm, we can align the 3D models by considering each complete model as a single point cloud. However, in a real scenario, the alignment error is not accurate, which affects the quantification of surface geometry changes. In the case of restoration of an object, if the object has induced changes only on a known part of the surface, the alignment error influence can be minimized. After the initial ICP algorithm, a second pass can be performed, considering only the unchanged part of the surface, as shown in Figure 2. Later, calculated transformation could be applied to the whole changed surface model. This is only to avoid alignment error and confirm the correctness of the proposed geometry comparison method [21]. However, when the object has a change on the whole surface, especially concerning an outdoor monitoring or even the chemical treatment or cleaning of the object’s surface [22], having no reference to be considered as no change, the entire 3D model must be aligned by using the ICP algorithm. There is a small possibility of avoiding alignment error, even from the concerned monitoring period, based on the expertise of the conservation scientists: a certain time interval can be considered as involving no change for some object’s material.

### 2.2. Global Geometry Analysis

In this section, the occurrence of surface changes was assessed by comparing both the target and source models obtained over time. After alignment of the models, the global change of the surface geometry over time was quantified. We calculated the changes in the geometry of the object for each surface point and its respective neighborhood points’ displacement behavior.

#### 2.2.1. Point to Point Absolute Distance (P2P)

**Figure 3:**
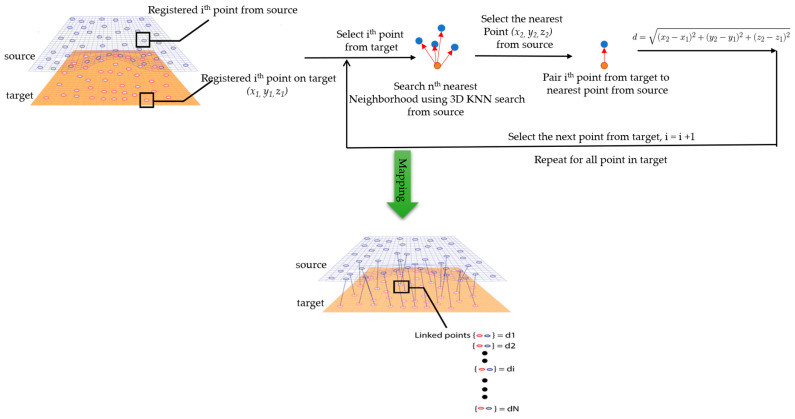
Sketch of the P2P distance analysis.

The comparison of the 3D models of the object was performed to assess the global change in the surface geometry. The analysis of the global change can help to identify roughly what changed on the surface. In this section, the absolute 3D distance from each surface point of the target to the nearest surface point on the source is calculated as shown in Figure 3. Based on the minimum and maximum values obtained, a color map is generated to represent the quantification of change on the target surface.

#### 2.2.2. Point-to-Point Vector Distance (P2P_Direction)

**Figure 4:**
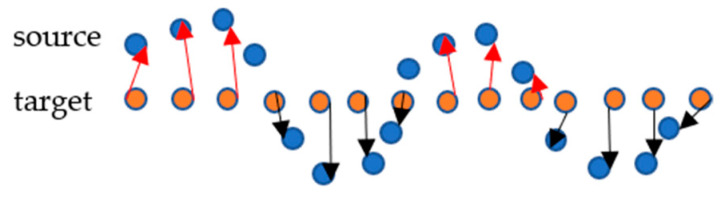
Direction of change distance calculation.

In the absolute distance calculation, the information was not clear enough regarding the direction of the change with respect to the target, as shown in Figure 4, with red (shifted up) and black (shifted down) arrows. Additionally, in the analysis, the conservators were interested to see whether the surface points are going up (deposit) or down (material loss).

In the direction distance analysis, the dot product of the normal vector (*n*) of each point on target was computed with the displacement vector (*d*) to each nearest point on the source. The normal vectors were calculated for each surface point of the target, creating a neighboring plane among its neighbor points, towards the direction to the 3D scanner for the necessary measurement device. The dot product between the two vectors gave a sign information to the change, considering the target as a reference of the change. The direction of change is an important factor that helps us to analyze the change in the surface to identify the surface points and whether it is a loss or a deposit. However, in the case of a known restoration process where gap filling is performed [21], P2P absolute distance calculation will work efficiently.

#### 2.2.3. Point-to-Point along Normal Vector (P2P_AlongNV)

**Figure 5:**
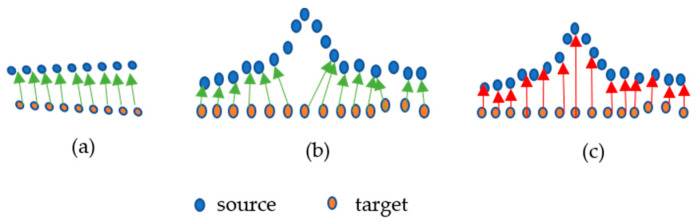
Scenarios (**a**). Smooth (**b**). Non smooth and (**c**). Actual of finding the corresponding changed point from target to source.

In the above distance calculation method, the consideration of the nearest point may not always correspond to the actual change point on the source, especially when it comes to the unsmooth and curvy parts of the surface. A scenario is presented in Figure 5. The consideration of the nearest point will work if the surface changes linearly, as shown in Figure 5a, but in Figure 5b, it will not correspond to the actual change point to the target. In the figure, the consideration of the changed point is shown with a green arrow and the actual changed point is shown with red arrow in Figure 5c.

**Figure 6:**
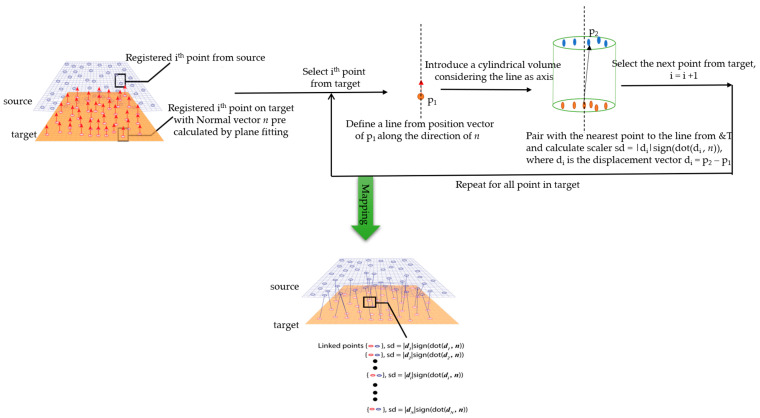
Sketch of the P2P_AlongNV distance calculation.

To avoid the corresponding change point consideration, in the analysis, a strategy was adopted whereby the corresponding change point was considered along the normal vector of the selected point on target to source. A line was drawn along the direction of the normal vector via the selected point from target, and the distance was computed within a specified cylindrical volume on source to the line. The nearest point to the line was considered as the corresponding changed point from source for each selected point on target. After finding the corresponding nearest point to source, the displacement vector was computed with the normal vector of target model. To optimize the search for a nearest point to the line, a user-defined radius was set. The working principle for the P2P_AlongNV distance calculation method is shown in Figure 6. The search will retrieve a zero value if the nearest point cannot be found within the specified region. In this case, when the search retrieves a value of zero, either the search radius can be increased for those points, or it can be presented as zero if it cannot find a point after the increase in the search radius.

#### 2.2.4. Point-To-Point Projection along Normal Vector (P2P_ProjectionAlongNV)

**Figure 7:**
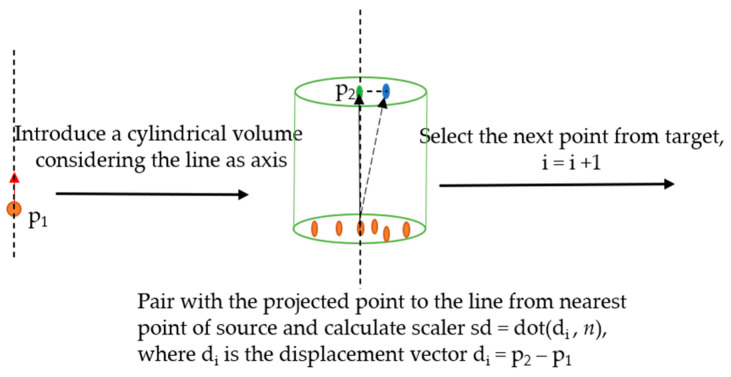
Sketch of the P2P_ProjectionAlongNV distance calculation.

In this section, the P2P_AlongNV was slightly modified by projecting the considered nearest point to the line along the normal vector, as shown in Figure 7. The corresponding change point was considered on the line, and the displacement vector was computed from the normal vector to quantify the change and its direction with respect to the target.

### 2.3. Local Geometry Analysis

**Figure 8:**
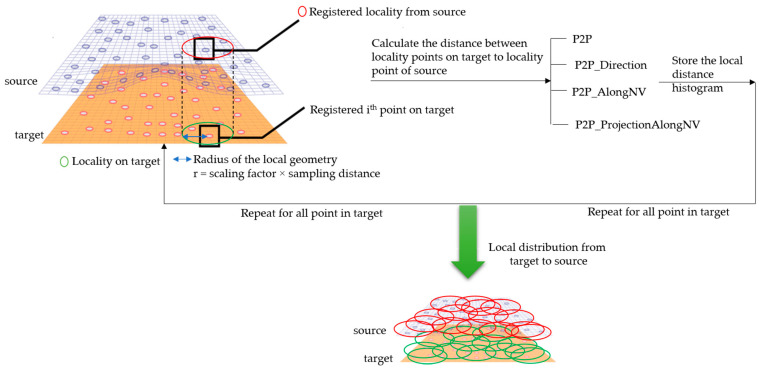
Sketch of the local neighborhood distance analysis.

The local geometry analysis is based on the optimized k-neighborhood points and computing the distances of four types, as mentioned above, with the source for each point on target. For each point on target, the nearest point was computed up to a radius (*r*) of the scaled average point-to-point distance of source, with consideration of the local neighborhood change analysis. The local distribution as shown in Figure 8 is the basis for a further analysis of the change information locally and grouping the data based on the similarity of their behavior.

### 2.4. Segmentation

In this section, a change-based segmentation is proposed to train the method to automatically detect the types of changes that occurs on the surface over time. The respective distances of the k-neighborhood points were calculated for each point on the target model and stored as the local distance behavior in histograms. Then, using the calculated local neighborhood histograms for each point, the calculated data were fit to the kernel distribution curve [23]. As the fluctuation of the local neighborhood distance histogram produced by the data is very irregular, the random and discrete kernel distribution can sum up the component smoothing functions for each data value to produce a smooth, continuous probability curve [24,25].

The estimation of kernel density function, f_n_, for given n number of points with density, f, is defined as below:

(1) $$f_{n}\;\left(x\right)=\sum^{\;}n_{j}=1\;K\;\left\{\left(x-{\;X}_{j}\right)\;h\;\right\}\;n\times h$$

here K and h represent the kernel function and the smoothing parameter or window width, respectively.

In Equation (1), the value of the window width, h, can be set by using Silverman’s (1986) recommendation [26], which uses a default width of the DATA PLOT as follows:

(2) $$0.9\times \min\left(s\;,\;\frac{\mathrm{IQR}}{1.34}\right)\times n-\frac{1}{5}$$

In Equation (2), s is the sample standard deviation, and IQR is the sample interquartile range.

The selection of the optimal window width depends on the underlying estimated function, and it works reasonably good for a wide variety of distribution.

The analysis showed that the local neighborhood distance histograms obtained are normally distributed in this work. Since the underlying data are normally distributed according to Silverman’s DATA PLOT, the optimal width is calculated as follows:

(3) $$1.06\times s\;\times n-\frac{1}{5}$$

where n is the number of points in the raw data, and s is the sample standard deviation of the raw data.

In the proposed approach, several parameters were considered, with the calculated local distance histogram as an input to the segmentation method, as shown in Table 1. Moreover, several user-input threshold values were incorporated into the segmentation method, as shown in Table 2, to make the grouping based on the 3D model fitting and quantitative analysis of the amount of change based on the size of the object. The bandwidth for each category of change segmentation was assigned based on the sampling distance and noise obtained at each of the point clouds from both stages of data. However, in a real scenario, as mentioned in Section 1, the data collected from CH surfaces are not structured and supervised. They also contain thermal noise from the detector of the 3D scanner detector. To overcome the noise from the surface and make the method insensitive to noisy data, the surface noise was parametrized, and conditions were set based on the obtained histogram parameters.

The conditions were set to the parameters considered in Table 1 and Table 2 for change-based segmentation.

The parameters were defined for the analysis, as in Table 3.

### 2.5. Visualization

**Figure 9:**
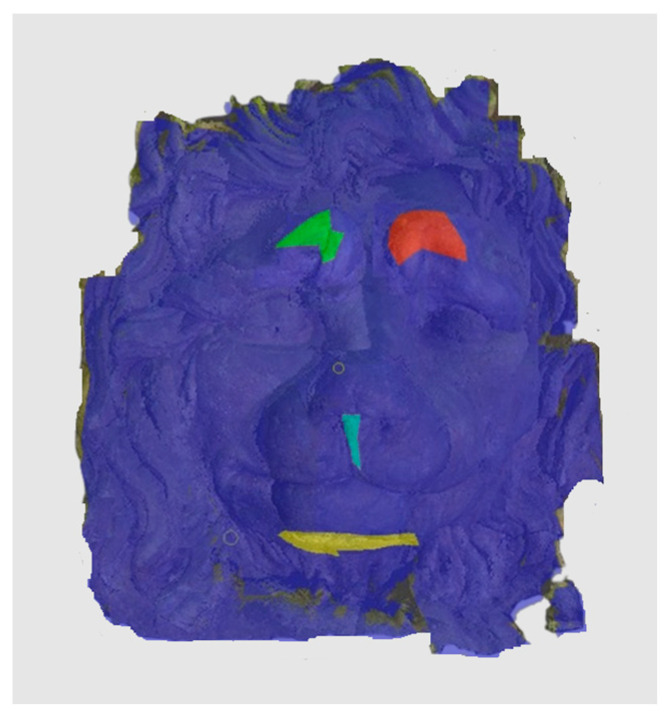
Exemplary visualization of detected changes on the surface for object originated from Figure 2.

To represent the results, after grouping the surface points with the respective type of change, a color map was generated on the target point cloud to represent the same on the surface. In the visualization section, the colors for each change type were specified and we set the colors to the target model to make it an easier and more user-friendly application. In the analysis, the colors chosen for each identified change were assigned, as shown in Table 4. The visualization on the surface is presented in the tables, with obtained results from the segmentation method for each type of change mentioned in Table 3. An exemplary model, as described in Figure 2, is presented in Figure 9, and identified changes are shown on the surface with the specific colors for each category of change.

## 3. Results and Analysis

Two case studies were considered for the analysis. For Case Study I, the data were collected by using a structured light-based custom-designed 3D scanner [27] developed at the Faculty of Mechatronics at Warsaw University of Technology, Poland. This device has a maximum permissible error of 0.25 mm. For Case Study II, the 3D datasets considered were from the PRESIOUS project [28]. The postprocessing of the data for the proposed method was written in FRAMES, which was also developed at the Faculty of Mechatronics, Warsaw University of Technology, Poland, and written in C++ [29].

### 3.1. Case Study I

#### 3.1.1. Results of Simulated Data

**Figure 10:**
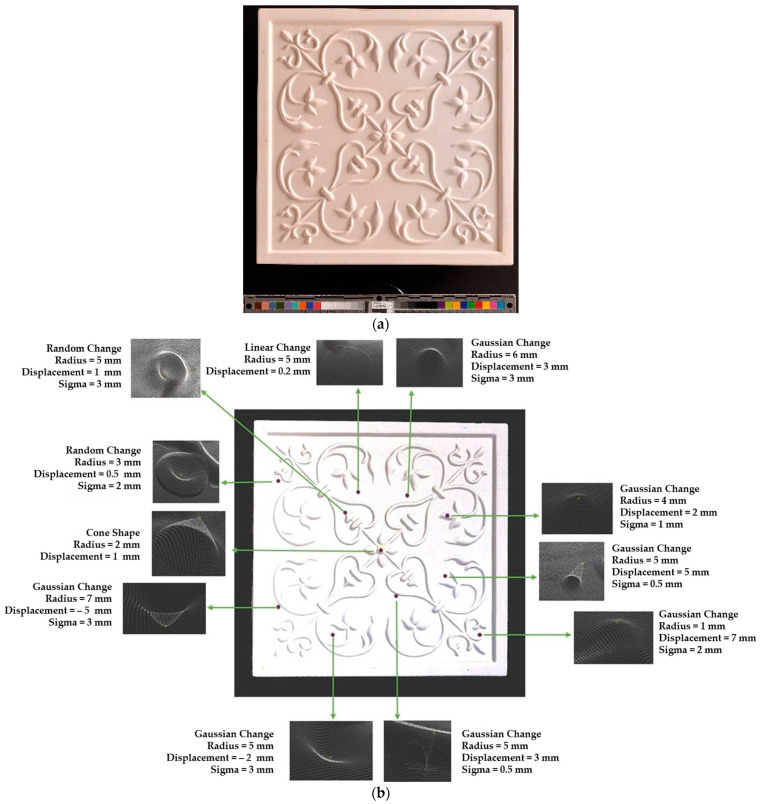
Tile reconstruction; ceramic; 220 × 220 × 45 mm. Owner: Academy of Fine Arts in Warsaw, Poland. (**a**) Original state of the tile and (**b**) changes introduced manually to the original surface.

To verify the appropriateness of the proposed segmentation method, a mock-up of a ceramic tile of size 220 × 220 × 45 mm^3^ for a tile stove was prepared by a group of restorers, as shown in Figure 10a. The approximate depth of relief of the tile was 4 mm. The original state of the model was scanned to obtain the target model. The captured data were filtered out from the outliers and external noise based on a Hausdroff distance calculation, and we removed faraway points based on a threshold input distance. The complete model was obtained after stitching each point cloud, using the ICP algorithm. At the beginning of the analysis, to assess the ground truth of Table 3 and avoid cross-time alignment error, several manual changes were made with known values on the original surface. The changes were introduced on the surface points to prepare the source model as shown in Figure 10b, and the obtained results with the input parameters as shown in Table 5 are shown in Table 6.

The results from the simulated surface, as shown in Table 6, for the proposed method, and specifically from the global geometry analysis of P2P, the negative and positive displacement cannot be predicted. However in the P2P_Direction, P2P_AlongNV, and P2P_ProjectionAlongNV global distance calculation, the direction of change is detectable. And from the segmentation analysis, the locations of the changes made and their impact on its local geometry were detected more clearly. In this case study, the segmentation approach of four different distance calculations did not show much difference, as the manual changes were made precisely and smoothly on selected points.

#### 3.1.2. Results of Real Scenario

**Figure 11:**
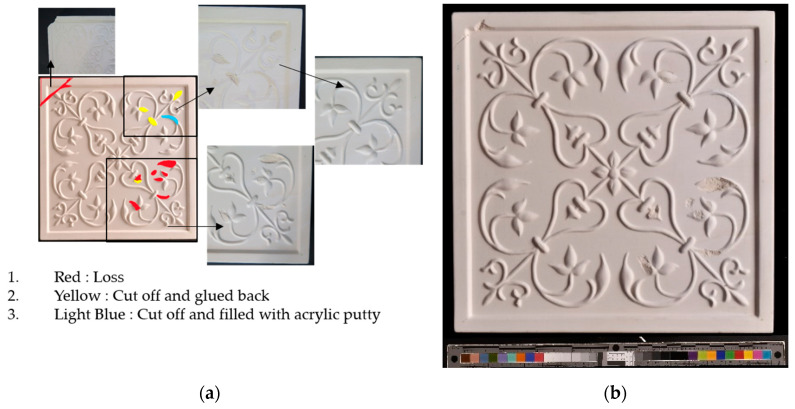
(**a**) Changes made to the tile; (**b**) the changed tile.

A tile was considered to represent the restoration problems encountered with sculptures and other ceramics. Several changes were introduced to the tile, such as loss, gluing, displacement of the decorations, gluing of a large piece of the object, and gap gilling with acrylic putty to achieve the reconstruction of the tile, as shown in Figure 11a. The original state of the tile is shown in Figure 10a. Figure 11b shows the outcome after the mechanical changes were made on the tile.

**Figure 12:**
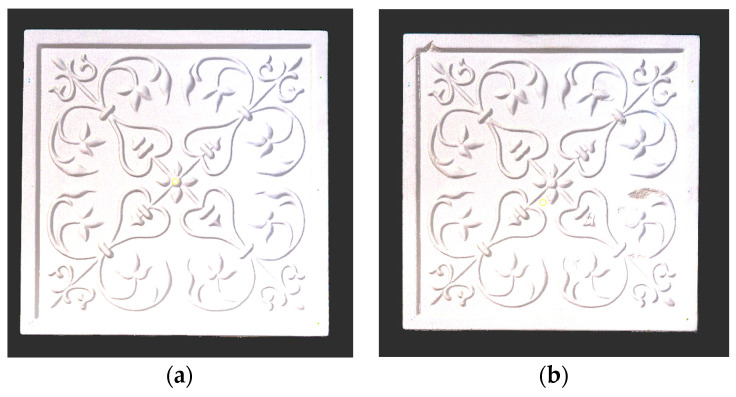
Three-dimensional models of the original surface (**a**) and changed surface (**b**) of Case Study I.

For a quantification of the induced change in the real scenario, the analysis had to register both the target and source models in one time frame, using the ICP algorithm mentioned in Section 3.1.1. To overcome the alignment error and places of changes made along with the noise, the Nonchanged Threshold (*N_t_*) described in Table 2, with reference to the unchanged part of the surface, was set. The input parameters to the segmentation method were set as shown in Table 5. The models obtained before and after are shown in Figure 12 and the calculated results are presented in Table 7.

The results from the ceramic tile were verified by a group of restorers and the correctness of the method was justified via the locations of damage and displacement on the surface, which could not be detected in a global geometry analysis. The results showed a loss of more than 2 mm of material where parts of the surface were damaged. In the analysis, we detected the misalignment of gluing as a deposit of less than 0.5 mm to the surface, indicating that, while gluing back the parts, they were not placed properly, or the thickness of glue caused the displacement of the decorations. The part of the surface that was filled with acrylic putty showed a displacement on the surface as the filling was slightly tilted in relation to the original shape. Moreover, during the removal of a corner of the tile for investigation reasons, a very small part of the ceramic was lost. The gluing was performed correctly, but the loss was not filled with putty; this part was recorded during the scanning process and noted as a loss in the analysis. The method can be claimed as correct in cases when a part of the surface remained unchanged, and the user can consider it as a reference to set the threshold *N_t_*. However, this practice is not suitable with an increase in the threshold *N_t_*, as this would result in losing some information due to both the minor deposit and the loss.

### 3.2. Case Study II

**Figure 13:**
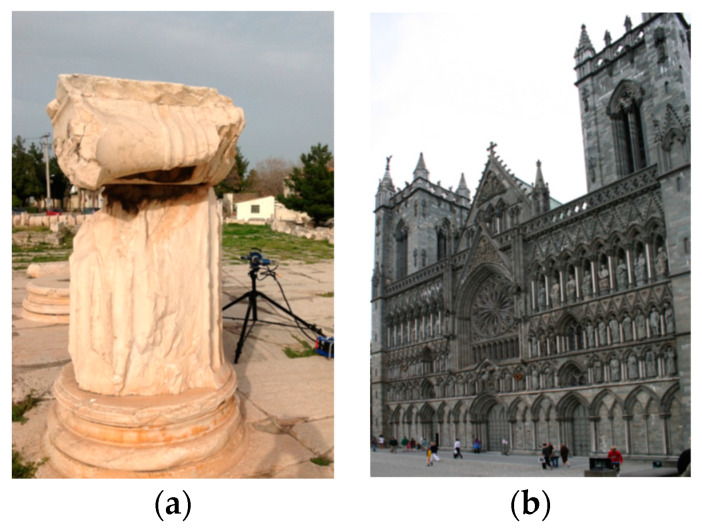
The two CH monuments considered for study: (**a**) The Sanctuary of Demeter and (**b**) Nidaros Cathedral in Trondheim, Norway.

The second case study considered monitoring over time. Several datasets were considered to test the method from the PRESIOUS project [28]. The monument sites that were studied during the project work are shown in Figure 13.

Several datasets were considered for the analysis, as shown in Table 8, from two sessions. During the project work, stone slabs were used for accelerated erosion experiments with the treatment of various chemicals and monitored over time.

The results show the erosion and loss of material from the stone slabs over time. The 3D models for Case Study II were obtained, as shown in Table 9, from the PRESIOUS project. Two different time interval models were registered in one time frame, using the ICP algorithm.

The analysis was carried out on each considered dataset with two different time measurement to assess the changes that occurred during the erosion process, with the values of parameters shown in Table 10. The results and a detailed discussion of the changes from chemical reactions (see Appendix A) are provided below. The calculated results obtained from the analysis and the corresponding surface point count information for each considered datasets are shown in Table 11, Table 12, Table 13, Table 14, Table 15, Table 16, Table 17, Table 18, Table 19, Table 20, Table 21 and Table 22.

It is difficult in an actual case study to determine which parts of the surface undergo no change or a minor deposit and loss as during the monitoring period there are no physical markers used as a reference. In this case study, the Nonchanged Threshold could not be increased as the part of the surface that remains unchanged over time was unknown. In addition, the mass loss and mean erosion calculations showed that the Nidaros Bad stones underwent a greater loss compared to Nidaros Good and Marble, and the same can be seen in the proposed analysis. Additionally, the point count analysis to measure the surface area with loss and deposit showed that Nidaros Bad stone slabs had a loss on a greater part of the surface compared to Nidaros Good. However, the marble slabs also showed a loss on a greater part of the surface, yet the quantification of loss was smaller than for Nidaros Good. The datasets of Nidaros Bad showed a total loss of material from the surface, as shown in Table 17 and Table 19. Nidaros Good showed more loss than the marble stone slabs. The analysis can be easily interpreted and claimed to be correct for any mechanical changes during the restoration process. However, in real-case monitoring, such as chemical weathering or physical/mechanical weathering, as performed in the PRESIOUS project, these are unpredictable.

## 4. Conclusions

In this work, an analysis method with two or more change stages for 3D geometrical data was developed. The 3D analysis of geometrical changes was performed by comparing the local geometry of two phases of surface information. This provides information about the behavior of geometry changes and segments the surface into areas with similar characteristics. The computational analysis of each surface point and its respective neighboring points’ behavior can provide the local geometry changes, comparing more than one phase of data and leading to a better understanding of the changes over time on CH surfaces.

In the distance calculation, the focus was on direct comparison of each surface point rather than meshing the surface to assess the direction of change. The P2P computation method provides an overall good visualization of the change parts on the surface failing to provide information about the direction of change. The P2P_Direction method works well in smooth and slightly eroded surfaces, providing the change direction as on the simulated surface for Case Study I, whereas the P2P_ProjectionAlongNV showed a promising result, providing more information irrespective of the surface smoothness and noisy data. The P2P_AlongNV method computes for a local distance between the two points along the normal vector direction but fails when the corresponding change point is far away from the line along the normal vector. The P2P_ProjectionAlongNV method provides additional projection confidence for each computation of the corresponding change point along the normal vector line providing better region segmentation. However, for simple deformations (in Case Study I), the P2P_Direction method provided an acceptable result, but showed a minor deposit where gluing was performed, whereas the P2P_AlongNV and P2P_ProjectionALongNV showed major deposits with the same value of deposit threshold, which agrees with the expectations. The P2P_AlongNV and P2P_ProjectionALongNV methods better quantified the change as P2P and P2P_Direction consider the immediate neighboring point from the compared dataset as a corresponding change point. For Case Study II, for the datasets of NGL2, NBL1, and NBL2, as shown in Table 15, Table 17, and Table 19, the distance calculation method P2P_ProjectionAlongNV provided more information about the major material loss with a better visualization. To sum up, the P2P_ProjectionAlongNV method allows for the visualization of changes most in line with the expectations of art conservators and is recommended for the assessment of changes in the surface of cultural heritage objects.

However, the proposed method is sensitive to the initial cross-time alignment of consecutive datasets. The aim of this work was to assess and classify the changes of surface geometry of CH objects from two-time intervals. However, the cross-alignment of the two models is an open issue when the entire surface has a change over time. In the analysis, we tried to solve the alignment issue up to a certain point by introducing the Nonchanged Threshold, but it is hard to claim the same when changes occur on the entire surface and there is no reference point with no change. The results should help improve the 3D documentation of the quantification of surface changes of CH objects over time. The analysis and its correctness were more promising in Case Study I than Case Study II, due to the lack of a reference with no change. The proposed analysis and grouping method could help lay users or conservators to analyze and present their work more clearly during restoration. The analysis can access the details of each surface point’s behavior and show how it is changing over time. The method is automated and insensitive to the noise of the input data. The user can set the threshold value of deposit, loss, and alignment based on the registration and easily access the surface change information. The colormap visualization of each change category makes the proposed method very convenient and user-friendly. This analysis is not limited in terms of the composition or size of the object, thus making the method more flexible and reliable.

In this paper, the measurement was performed on surface geometry change segmentation. However, displacement or deformation of material, changes in the shininess of a CH surface, color, or spectral information changes should be taken into consideration. This work can be extended to multimodal image data. The proposed analysis will be helpful for tracking down changes of surface texture, displacement, and color information over time. In the future, this segmentation approach will be tested on multimodal image data, considering reflectance imaging transformation, digital image correlation, and multispectral imaging.

## Figures and Tables

**Table 1:** Considered input parameters to the segmentation method.

Parameters	Unit	Description	Visual Paradigm
Sampling distance (*sd*)	Millimeter	The average point-to-point distance of the input data, i.e., resolution of the 3D scanner, is considered as the sampling distance.	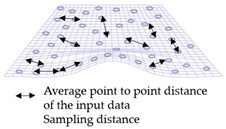
Knn_Count (*knn*)	Integer	The total number of points, i.e., size of neighborhood inside each spherical neighborhood considered for the local geometry analysis, is termed Knn_Count.	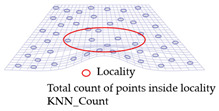
Bandwidth (*B_w_*) and Mean (*m_u_*)	Real	The bandwidth/number of bins that illustrate the appearance of the local distance histogram. The Bandwidth is calculated as in Equation (3). The mean of the histogram is the average of the values in the local distance histogram. Both the bandwidth and the mean are normalized to the resolution of the input data to make the segmentation method more independent of the input data.	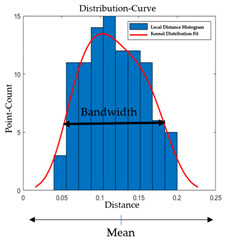
Noise (*N*)	Real	The noise is basically calculated as the plane fitting error for the input data. The noise is calculated by using the random sampling of the input data. The randomly selected points were fitted to a best-fitting plane, considering the neighboring points up to a given radius of *scaling factor × sd*. The scaling factor for the analysis was set to 5; however, the user can scale it based on the density of the data. From randomly chosen points from the surface, the plane fitting error was calculated in terms of RMS distance from the considered points to the calculated plane. In this case, the noise was averaged from both the original and the changed surface. To make the method more independent of the resolution of the input data and the amount of noise, it was normalized with the calculated sampling distance.	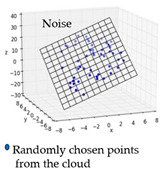
Error 1 (*e_1_*) and Error 2 (*e_2_*)	Millimeter	From each locality and its corresponding local distribution, the plane error was calculated, and we inspected the behavior of those errors for the segmentation method. Both the original surface and the changed surface locality were considered for RMS distance calculation from the points to the local plane. However, this RMS distance calculation is confined to the local distribution, unlike the noise from the entire surface.	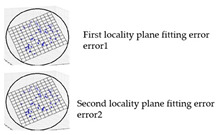

**Table 2:** User input thresholds to the segmentation method.

Threshold	Description
Deposit/Loss Threshold (*D_t_*)/(*L_t_*)	The deposit/loss threshold is used to define the amount of deposit/loss to/from the surface. This user input will quantify this as minor or major according to the change with respect to the size of the object. A deposit/loss less than the size of the deposit/loss threshold will be presented as minor while one greater than the threshold will be considered a major deposit/loss on the surface.
Nonchanged Threshold (*N_t_*)	This threshold was considered based on the alignment of the 3D models and the accuracy. The alignment threshold is a ratio of normalized noise, for which a default value is set to 1. However, the user can increase the value in certain cases with known reference to an unchanged part of the surface. In some cases, based on user expertise of the object material and the considered time interval, an unchanged threshold can be set to a particular value that will provide more accuracy for the quantification of changes.

**Table 3:** Ground truth based on the histogram parameters.

Change Types	Local Naming	Parameter’s Behavior
Type 1	No Change	$B_{w}<\frac{N}{knn}\times N_{t}\;\&\&\;m_{u}<\frac{sd}{knn}\times N_{t}$
Type 2	Minor Change (Deposit) Equal Unequal Linear Nonlinear	$m_{u}\leq D_{t}\;\&\&\;m_{u}>0$ *B_w_* < *N* *B_w_* > *N* (*e_1_*, *e_2_*) < *N* (*e_1_*, *e_2_*) > *N*
	Minor Change (Loss) Equal Unequal Linear Nonlinear	$m_{u}\geq L_{t}\;\&\&\;m_{u}<0$ *B_w_* < *N* *B_w_* > *N* (*e_1_*, *e_2_*) < *N* (*e_1_*, *e_2_*) > *N*
Type 3	Major Change (Deposit) Equal Unequal Linear Nonlinear	$m_{u}>D_{t}\;\&\&\;m_{u}>0$ *B_w_* < *N* *B_w_* > *N* (*e_1_*, *e_2_*) < *N* (*e_1_*, *e_2_*) > *N*
	Major Change (Loss) Equal Unequal Linear Nonlinear	$m_{u}<L_{t}\;\&\&\;m_{u}<0$ *B_w_* < *N* *B_w_* > *N* (*e_1_*, *e_2_*) < *N* (*e_1_*, *e_2_*) > *N*
Type 4	Unknown	Other behavior of parameters.

**Table 4:** Assigned color code and number for each category of change.

Change Types	Assigned Code	Assigned RGB Code/Color		
Type 1	0	(0, 0, 255) 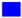		
Type 2	+1 (Deposit)	(0, 128, 255) 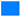 Equal	(0, 255, 255) 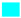 Linear	(153, 255, 255) 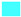 Non-linear
	−1 (Loss)	(255, 255, 153) 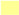 Equal	(255, 255, 0) 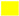 Linear	(255, 128, 0) 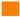 Non-linear
Type 3	+2 (Deposit)	(153, 255, 153) 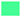 Equal	(0, 255, 0) 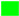 Linear	(0, 102, 0) 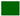 Non-linear
	−2 (Loss)	(255, 102, 102) 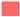 Equal	(255, 0, 0) 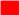 Linear	(153, 0, 0) 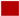 Non-linear
Type 4	X	(102, 0, 0) 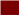		

**Table 5:** Calculated and user input parameters for Case Study I.

Datasets	*N* (mm)	*sd* (mm)	Normalized Noise *N/sd*	*N_t_* (Scaled by N/sd)	*D_t_* (mm)	*L_t_* (mm)
Simulated data	0.015	0.098	0.15	1	1	1
Real scenario	0.014	0.010	0.14	100	0.5	2

**Table 6:** Correctness of the ground truth table.

Method	Global Geometry Analysis	Segmentation
P2P	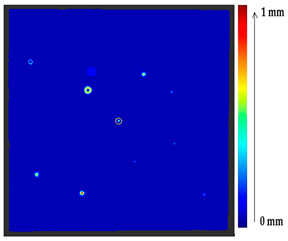	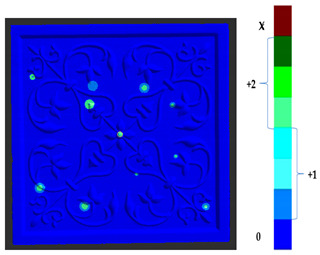
P2P_Direction	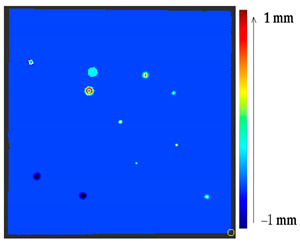	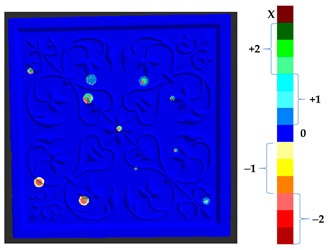
P2P_AlongNV	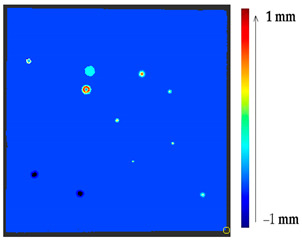	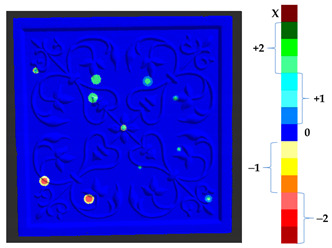
P2P_ProjectionAlongNV	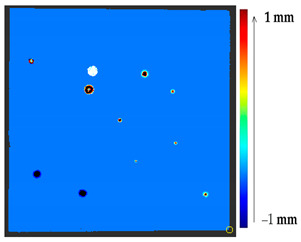	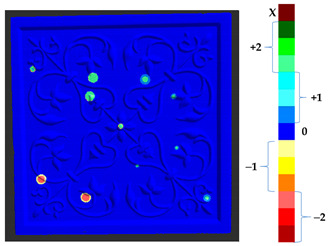

**Table 7:** Correctness of the ground truth table with Nonchanged Threshold.

Method	Global Geometry Analysis	Segmentation
P2P	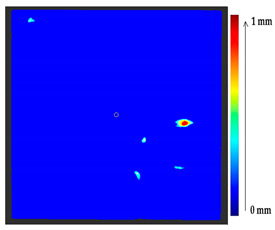	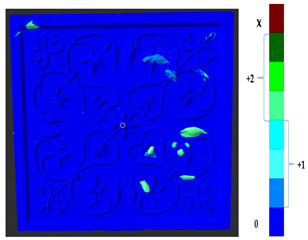
P2P_Direction	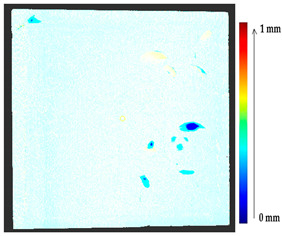	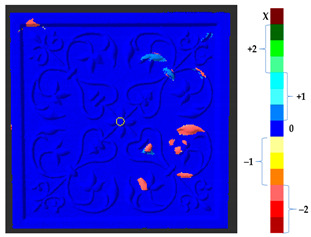
P2P_AlongNV	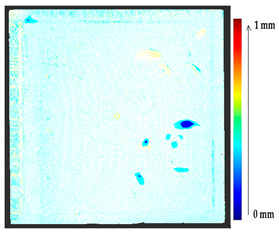	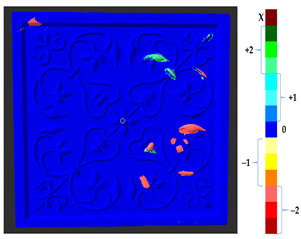
P2P_ProjectionAlongNV	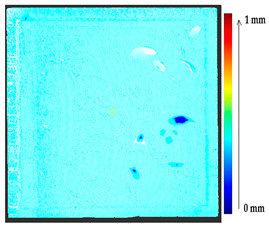	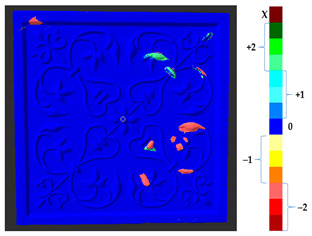

**Table 8:** Stone-slab labeling and two chosen sessions of data collection.

Considered Stone Slabs for the Analysis and Monitoring Period		
Stone Slabs (Naming from [30])	Session 1	Session 2
Elefsis Large 03(EL3)	2015-01-12	2015-05-15
Elefsis Small 01 (ES1)	2015-01-12	2015-05-15
Nidaros Good Large 02 (NGL2)	2015-01-12	2015-05-15
Nidaros Bad Large 01 (NBL1)	2015-01-12	2015-12-04
Nidaros Bad Large 02 (NBL2)	2015-01-12	2015-05-15
Nidaros Good Small 01 (NGS1)	2015-01-12	2015-05-15

**Table 9:** Three-dimensional models of the considered stone slabs from two different times.

Stone Slabs	Original Surface	Changed Surface
	Session 1	Session 2
EL3	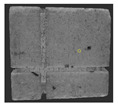	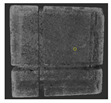
ES1	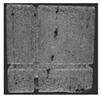	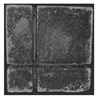
NGL2	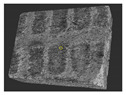	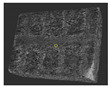
NBL1	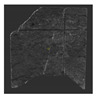	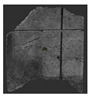
NBL2	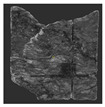	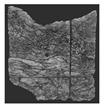
NGS1	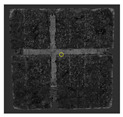	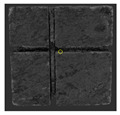

**Table 10:** Calculated and user input parameters for Case Study II.

Datasets	*N* (mm)	*sd* (mm)	Normalized Noise *N/s*	*N_t_* (Scaled by *N/s*)	*D_t_* (mm)	*L_t_* (mm)
EL3	0.006	0.072	0.09	1	0.1	0.2
ES1	0.006	0.072	0.08		0.1	0.2
NGL2	0.007	0.062	0.10		0.15	0.5
NBL1	0.007	0.056	0.12		0.25	1
NBL2	0.008	0.065	0.12		0.25	1
NGS1	0.008	0.070	0.11		0.15	0.5

**Table 11:** Analysis and results for the dataset of EL3.

Method	P2P	P2P_Direction	P2P_AlongNV	P2P_ProjectionAlongNV
Global Geometry Analysis	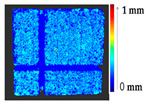	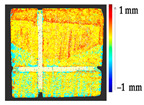	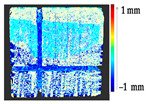	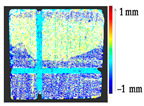
Segmentation	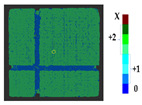	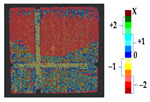	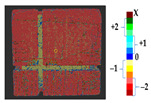	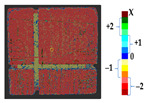

**Table 12:** Analysis of surface points count on the surface of EL3.

Segmentation	Total Point Count	No Change	Minor Change		Major Change		% of Surface with Deposit	% of Surface with Loss
			Deposit	Loss	Deposit	Loss		
P2P_Direction	976,587	1	363,788	496,653	13,368	115,978	37.25	62.74
P2P_AlongNV		1	261,220	393,974	4464	316,928	27.20	72.80
P2P_ProjectionAlongNV		557	267,661	407,043	5768	295,558	27.01	72.95

**Table 13:** Analysis and results for the dataset of ES1.

Method	P2P	P2P_Direction	P2P_AlongNV	P2P_ProjectionAlongNV
Global Geometry Analysis	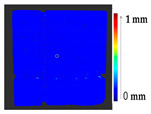	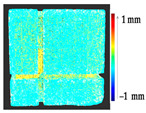	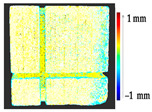	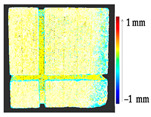
Segmentation	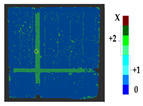	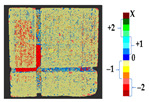	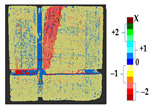	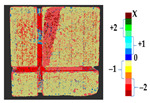

**Table 14:** Analysis of surface points count on the surface of ES1.

Segmentation	Total Point Count	No Change	Minor Change		Major Change		% of Surface with Deposit	% of Surface with Loss
			Deposit	Loss	Deposit	Loss		
P2P_Direction	854,730	0	286,411	473,175	73,094	22,050	42.06	57.94
P2P_AlongNV		0	159,543	310,138	159,421	225,628	37.31	62.69
P2P_ProjectionAlongNV		5	299,863	50,2268	16,905	35,689	37.06	62.94

**Table 15:** Analysis and results for the dataset of NGL2.

Method	P2P	P2P_Direction	P2P_AlongNV	P2P_ProjectionAlongNV
Global Geometry Analysis	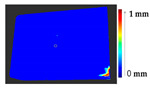	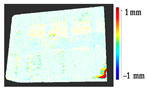	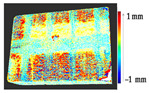	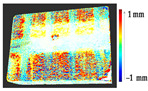
Segmentation	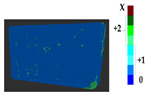	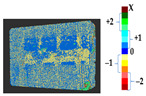	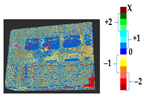	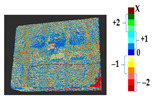

**Table 16:** Analysis of surface points count on the surface of NGL2.

Segmentation	Total Point Count	No Change	Minor Change		Major Change		% of Surface with Deposit	% of Surface with Loss
			Deposit	Loss	Deposit	Loss		
P2P_Direction	3,545,412	11	1,792,619	1,587,970	97,467	67,345	53.32	46.69
P2P_AlongNV		22	1,817,344	1,426,692	14,482	286,872	51.67	48.33
P2P_ProjectionAlongNV		872	1,828,897	1,437,406	15,926	262,311	52.03	47.98

**Table 17:** Analysis and results for the dataset of NBL1.

Method	P2P	P2P_Direction	P2P_AlongNV	P2P_ProjectionAlongNV
Global Geometry Analysis	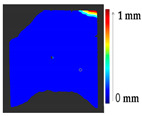	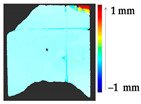	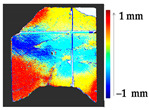	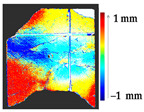
Segmentation	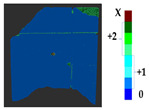	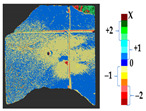	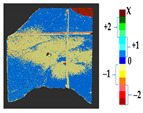	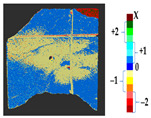

**Table 18:** Analysis of surface points count on the surface of NBL1.

Segmentation	Total Point Count	No Change	Minor Change		Major Change		% of Surface with Deposit	% of Surface with Loss
			Deposit	Loss	Deposit	Loss		
P2P_Direction	2,671,989	135	924,015	1,337,342	122,829	287,667	39.17	60.82
P2P_AlongNV		138	870,475	1,309,608	10,023	481,745	32.95	67.04
P2P_ProjectionAlongNV		2083	860,933	1,376,565	12,852	419,556	32.70	67.22

**Table 19:** Analysis and results for the dataset of NBL2.

Method	P2P	P2P_Direction	P2P_AlongNV	P2P_ProjectionAlongNV
Global Geometry Analysis	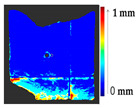	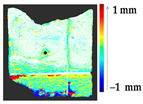	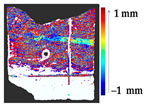	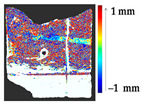
Segmentation	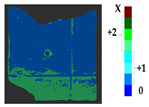	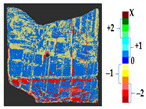	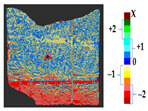	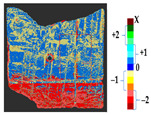

**Table 20:** Analysis of surface points count on the surface of NBL2.

Segmentation	Total Point Count	No Change	Minor Change		Major Change		% of Surface with Deposit	% of Surface with Loss
			Deposit	Loss	Deposit	Loss		
P2P_Direction	3,978,584	3	1,502,188	1,780,675	229,699	466,019	43.53	56.46
P2P_AlongNV		3	1,607,029	1,542,373	6891	822,280	40.57	59.43
P2P_ProjectionAlongNV		161	1,802,635	1,595,841	5951	573,996	45.46	54.53

**Table 21:** Analysis and results for the dataset of NGS1.

Method	P2P	P2P_Direction	P2P_AlongNV	P2P_ProjectionAlongNV
Global Geometry Analysis	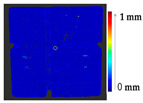	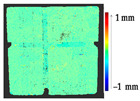	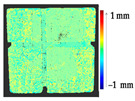	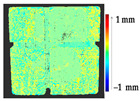
Segmentation	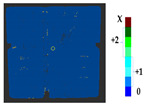	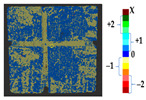	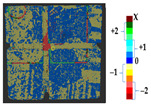	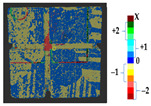

**Table 22:** Analysis of surface points count on the surface of NGS1.

Segmentation	Total Point Count	No Change	Minor Change		Major Change		% of Surface with Deposit	%of Surface with Loss
			Deposit	Loss	Deposit	Loss		
P2P_Direction	600,857	38	242,324	349,888	3677	4930	40.94	59.05
P2P_AlongNV		42	200,138	370,322	8787	21,568	34.78	65.22
P2P_ProjectionAlongNV		1218	201,266	369,771	8974	19,628	34.99	64.80

**Figure A1:**
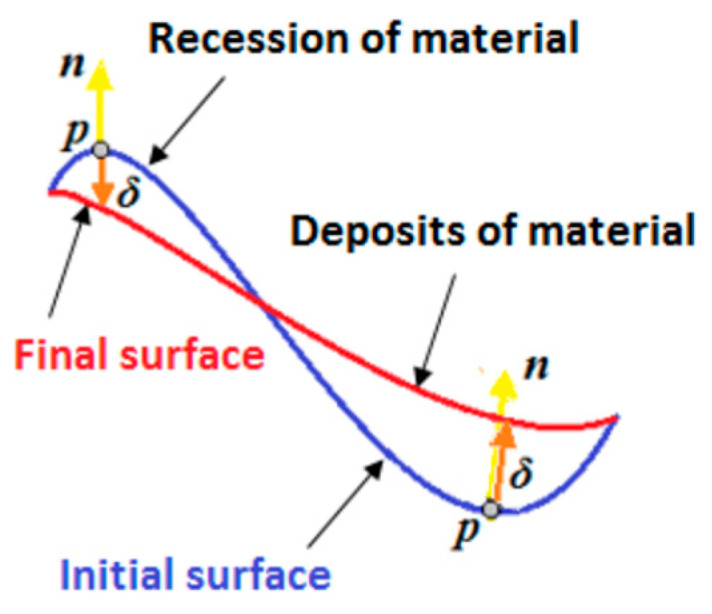
Modeling an erosion process on the surface of a stone.

## Data Availability

The data used for this study will be made public at the end of the project via CHANGE-ITN project website and its data repository and can be accessed on request.

## Appendix A

The documentation during the PRESIOUS work (Figure A1) and a calculation of the mass loss and mean erosion helped confirm the correctness up to a certain point, considering the alignment error. This analysis shows the loss and deposit on the stone’s surface due to several chemical phenomena.

**Table A1:** Stone slab material and associated experiments.

Considered Stone Slabs for the Analysis		
Stone Slabs	Material	Experiment
EL3	Pentelic Marble	H_2_SO_4_ + HNO_3_(aq) Acid
ES1	Pentelic Marble	H_2_SO_4_(aq) Acid
NGL2	Grytdal Soapstone	Salt
NBL1	Grytdal Soapstone	Freeze-Thaw
NBL2	Grytdal Soapstone	Salt
NGS1	Grytdal Soapstone	Outdoors/Trondheim

The acidic treatment performed during the project work on the slabs of EL3 and ES1 (marble) led to them undergoing a chemical reaction on the surface. During this chemical reaction with sulfuric acid (H_2_SO_4_), a crystallized gypsum formed on the surface, as shown below:

CaCO_3_+H_2_SO_4_ → CaSO_4_ + CO_2_ + H_2_O (A1)

CaSO_4_ + H_2_O → CaSO_4_ · 2H_2_O (A2)

The formation of gypsum, which crystallizes as a dihydrous gypsum that is 150 times better (2.1 g/L) than a salt, such as CaCO_3_, on the surface would appear as a deposit on the surface during the geometry analysis. The formation of crystalline gypsum CaSO_4_·2H_2_O during the acidic treatment can cause destruction of marble due to bigger volume of gypsum by 100% than CaCO_3_, and it can show a deposit for a certain period but may also show as a loss from the surface after some point [30].

The compound formed during the treatment with nitric acid (CaCO_3_) is characterized by a very high solubility, as follows:

CaCO_3_ + 2HNO_3_ → Ca (NO_3_)_2_ + CO_2_ + H_2_O (A3)

Due to the low solubility of calcium salts, nitrates are washed away by rainwater or migrates to the stone as a solution where it enters to the decomposition process which may cause in loss of marble mass.

A strong acid treatment, such as H_2_SO_4_ and HNO_3_, on the surface of the soapstone might absorb water due to its higher porosity and lead to a crack/hole and then a part falling off from the edges or weak parts of the stone slabs. However, it is very hard to draw conclusions about the change behavior of the chemical treatment on the stone slabs due to insufficient information. From a restorer’s point of view, it is good practice to keep a reference point for making calculations of deposit and loss. Moreover, it must be kept in mind at which stage of the destruction phase the investigation started. The time interval chosen for monitoring might not be proper to investigate some interesting changes for conservation studies and make preventive restoration decisions.

The freeze-and-thaw treatment performed on the soapstone (NBL1), which was performed at −5 °C, resulted in a crystalline form of ice. Ice, having a larger volume, exerts a pressure of 0.6 MPa at the chosen temperature [30]. The capillary crack on the corner of the stone slab of NBL1, as recorded during the first session of scanning, might get filled up with water. This capillary crack is more prone to the action of ice, which can cause a total loss from the edges of the stone slab. However, for this treatment, there was also no reference point of unchanged surface, as the freezing must be performed on the entire stone slab.

The monitoring of the outdoor environment on NGS1 reflects the consideration of several environmental factors, such as the action of changes in temperature, humidity, pressure, and changes in the climate of location from where the object belongs to. These external factors can lead to the disintegration of the stones into smaller particles during the monitoring period. This kind of monitoring loses the reference of the unchanged surface, leading to unpredictable changes in terms of loss/deposit.

**Table A2:** Changes in terms of mass of the selected stone slabs.

Stone Slabs	m_1_ (g)	m_2_ (g)	Δm (g)	Δm/m (%)
EL3	27.5131	27.4734	−0.0397	−0.14
ES1	27.5872	27.5156	−0.0716	−0.26
NGL2	160.5487	159.4037	−1.1450	−0.71
NBL1	168.8975	Unknown		
NBL2	188.9025	179.8329	−9.0696	−4.80
NGS1	20.6227	20.5998	−0.0229	−0.11

Mass measurements during the project work showed that the stone slabs suffered from erosion under the specific experimental conditions of chemical treatment, in the following order:

Marble ≈ Nidaros Good < Nidaros Bad (A4)

The salt effect (NGL2 and NBL2) was shown to be more dramatic than the freeze–thaw effects, which were quite close to the acidic effects, and Nidaros Bad was the most sensitive stone type. Table A3 gives estimations of erosion *δ* by volume and surface area computations: (a) cubic approximation and (b) surface area approximation; and V_1_ initial volume (session 1) and V_2_ initial volume (session 2) of the accelerated erosion period are presented.

**Table A3:** Estimated erosion by volume and surface area computation of the selected stone slabs.

Mean Erosion δ (mm) during Session 1 to Session 2						
Stone Slabs	V_1_ (cm^3^)	V_2_ (cm^3^)	ΔV (cm^3^)	S (cm^2^)	δ^(a)^ (mm)	δ^(b)^ (mm)
EL3	10.1391	10.1575	0.0184	29.7689	0.0065	0.0062
ES1	10.1510	10.1497	−0.0013	29.9718	−0.0005	−0.0004
NGL2	55.2833	55.1410	−0.1423	102.5810	−0.0164	−0.0139
NBL1	61.7922	Unknown				
NBL2	68.6979	66.6648	−2.0331	125.6727	−0.2040	−0.1618
NGS1	7.1294	7.1398	0.0104	24.6578	0.0047	0.0042

The mass loss and erosion during the chemical treatment, as shown in Table 10 and Table 11, could be identified from the proposed analysis.
